# Development and Validation of a Clinical Prediction Rule for Bacteremia among Maintenance Hemodialysis Patients in Outpatient Settings

**DOI:** 10.1371/journal.pone.0169975

**Published:** 2017-01-12

**Authors:** Sho Sasaki, Takeshi Hasegawa, Hiroo Kawarazaki, Atsushi Nomura, Daisuke Uchida, Takahiro Imaizumi, Masahide Furusho, Hiroki Nishiwaki, Shingo Fukuma, Yugo Shibagaki, Shunichi Fukuhara, on behalf of Japanese investigatOrs with Innovative Network for Kidney Disease: JOINT-KD

**Affiliations:** 1 Department of Healthcare Epidemiology, Kyoto University Graduate School of Public Health, Kyoto, JAPAN; 2 Center for Innovative Research for Communities and Clinical Excellence, Fukushima Medical University, Fukushima, JAPAN; 3 Division of Nephrology and Hypertension, Department of Internal Medicine, St. Marianna University School of Medicine, Kawasaki, JAPAN; 4 Office for Promoting Medical Research, Showa University, Tokyo, JAPAN; 5 Division of Nephrology, Department of Internal Medicine, Showa University Fujigaoka Hospital, Yokohama, JAPAN; 6 Division of Nephrology, Department of Internal Medicine, Inagi Municipal Hospital, Inagi, JAPAN; 7 Department of Immunology, Juntendo University School of Medicine, Tokyo, JAPAN; 8 Department of Nephrology, Chubu Rosai Hospital, Nagoya, JAPAN; 9 Department of Nephrology and Hypertension, Kawasaki Municipal Tama Hospital, Kawasaki, JAPAN; 10 Department of Nephrology, Toyohashi Municipal Hospital, Toyohashi, JAPAN; 11 Department of Nephrology, Nagoya University Graduate School of Medicine, Nagoya, JAPAN; 12 Department of Nephrology, Aso Iizuka Hospital, Iizuka, JAPAN; The University of Tokyo, JAPAN

## Abstract

**Background:**

To our knowledge, no reliable clinical prediction rule (CPR) for identifying bacteremia in hemodialysis (HD) patients has been established. The aim of this study was to develop a CPR for bacteremia in maintenance HD patients visiting the outpatient department.

**Methods:**

This multicenter cohort study involved consecutive maintenance HD patients who visited the outpatient clinic or emergency room of seven Japanese institutions between August 2011 and July 2013. The outcome measure was bacteremia diagnosed based on the results of blood cultures. The candidate predictors for bacteremia were extracted through a literature review. A CPR for bacteremia was developed using a coefficient-based multivariable logistic regression scoring method, and calibration was performed. The test performance was then assessed for the CPR.

**Results:**

Of 507 patients eligible for the study, we analyzed the 293 with a complete dataset for candidate predictors. Of these 293 patients, 48 (16.4%) were diagnosed with bacteremia. At the conclusion of the deviation process, body temperature ≥ 38.3°C, heart rate ≥ 125 /min, C-reactive protein ≥ 10 mg/dL, alkaline phosphatase >360 IU/L, and no prior antibiotics use within the past week were retained and scored. The CPR had a good fit for the model on calibration. The AUC of the CPR was 0.76, and for score CPR ≥ 2, the sensitivity and specificity were 89.6% and 51.4%, respectively.

**Conclusions:**

We established a simple CPR for bacteremia in maintenance HD patients using routinely obtained clinical information in an outpatient setting. This model may facilitate more appropriate clinical decision making.

## Introduction

Bacteremia is a common and serious condition worldwide and associated with a high mortality [1]. This condition is a particularly thorny problem in hemodialysis (HD) patients, since the morbidity and mortality of bacteremia in this population are extremely high compared to the general population [2–10]. Therefore, appropriate diagnosis and treatment are of great import in HD patients, as their outcomes can be quite poor.

Several clinical prediction rules (CPRs) with excellent predictive ability for bacteremia in the general population have been developed in recent years [11–14]. However, these CPRs include predictors that are difficult to apply to HD patients, such as serum creatinine, blood urea nitrogen, and electrolyte concentration, since these variables are known to be greatly affected by dialysis treatment. To our knowledge, no CPRs for bacteremia in HD patients have yet been established, a marked unmet need.

Two major differences have been noted with respect to bacteremia infection between maintenance HD patients and the general population. First, the incidence of bacteremia is much higher in maintenance HD patients than in the general population, as mentioned above. Previous cohort studies have shown that the incidence of bacteremia in maintenance HD patients is 10.40–18.98 per 100 person-years [2–7, 15], which is higher than the incidence of 0.216 per 100 person-years in the general population [1]. Further, the annual mortality due to bacteremia in HD patients is 100–300 times that in the general population. Even after adjustment for age, race, sex, diabetes status, and record errors, the mortality due to bacteremia is still 50 times higher in these patients than in the general population [8–10]. As such, bacteremia is considered a disease that is both extremely common and associated with serious outcomes in maintenance HD patients. Second, the pathogen type and etiology of bacteremia in maintenance HD patients differs from those commonly observed in the general population. The most frequently identified pathogen of bacteremia in the general population is *Escherichia coli* (*E*. *coli*), accounting for 22%-54% of cases [1, 16–21], with the urinary tract reportedly the most common route of infection, followed by the respiratory and gastrointestinal tracts [22]. In contrast, in maintenance HD patients, the most frequently identified pathogen of bacteremia is *Staphylococcus aureus*, accounting for 27%-39% of cases [6, 23, 24], with the most frequent routes of infection reportedly transdermal and trans-catheter sites.

Despite the substantial differences in the pathology and prognosis of bacteremia between HD patients and the general population, no CPRs have yet been established specifically targeting this at-risk group. In the present study, we developed and validated a new CPR for bacteremia specifically for maintenance HD patients.

## Materials and Methods

### Study Design

We conducted a multicenter retrospective cohort study of maintenance HD patients at six tertiary-care institutions, all of which receive patients on an emergency basis and all of which provide primary, secondary, and tertiary care; and one secondary-care institution that receives patients on an emergency basis and provides both primary and secondary care. All seven institutions are teaching hospitals. The present study was approved by the ethics committee of St. Marianna Medical University (No. 2713), and the study was conducted in accordance with the ethical standards of the Declaration of Helsinki. In the present study, the Department of Nephrology and Hypertension, St. Marianna University School of Medicine, connected anonymous data from the participating facilities. In addition, since all of the patient information analyzed in this study was retrospective, the consent of participants was not obtained.

In the present study, the Department of Nephrology at Chubu Rosai Hospital compiled anonymous data from the participating facilities. In addition, since all patient information analyzed in this study was retrospective, participants’ consent was not obtained. The study results are reported in accordance with the Transparent Reporting of a multivariable prediction model for Individual Prognosis Or Diagnosis (TRIPOD) statement [25].

### Participants

The inclusion criteria of the present study were as follows: consecutive HD patients who visited the outpatient department or emergency room between August 2011 and July 2013 and had blood drawn for cultures within 48 h from their initial arrival at the hospital. The exclusion criteria of this study were follows: under 18 years old; low frequency of HD (<1 time per week); combination of peritoneal dialysis; less than 2 weeks from the introduction of HD; and hospitalized patients referred from another hospital.

Among the participants who met the eligibility criteria, those with a complete dataset for candidate predictors were assigned to the derivation set. The set for internal validation was extracted using the bootstrap method.

We estimated that more than 10 cases with the outcome for each potential predictor in a multivariate model were needed to develop a clinical prediction model, based on common practice. Since we were attempting to create a CPR consisting of 5 item predictors, 50 outcomes were estimated to be necessary. The proportion of bacteremia in our study participants were estimated to 16%, based on the findings of previous Japanese studies conducted in the general population, which is thought to have a lower proportion of bacteremia than an HD population. We therefore estimated the required sample size to be a minimum of 320.

### Outcome Measures

The primary outcome measure was bacteremia diagnosed is based on the results of blood cultures at the time of the patient`s visit. The diagnosis of bacteremia was made when any bacteria not attributed to contamination were detected in a blood culture. Contamination was defined as follows: cases where only 1 of 2 sets of culture bottles was positive, or cases with detection of certain species of bacteria, such as diphtheroids, *Bacillus sp*., *Propionibacterium sp*., *micrococci*, *Corynebacterium sp*., and coagulase-negative *staphylococci*. Finally, an external consensus panel of two physicians well-trained in infectious diseases determined whether a culture was contaminated or not based on the above definitions and their clinical expertise.

### Candidate Predictors

Exhaustive variables already known to be predictors for bacteremia were selected from CPRs analyzed in an existing systematic review [11] and extracted by adding a search period through April 1, 2016, using the same search formula of MEDLINE via PubMed as a systematic review by Eliakim et al., as follows: ((predict[All Fields] OR predicting[All Fields] OR prediction[All Fields]) AND (("bacteraemia"[All Fields] OR "bacteremia"[MeSH Terms] OR "bacteremia"[All Fields]) OR (("blood"[Subheading] OR "blood"[All Fields] OR "blood"[MeSH Terms]) AND ("rivers"[MeSH Terms] OR "rivers"[All Fields] OR "stream"[All Fields]) AND ("infection"[MeSH Terms] OR "infection"[All Fields])))) AND ("2014/10/01"[PDAT]: "2016/04/01"[PDAT]) (12–14). The candidate predictors were then selected from among these exhaustive predictors through consultation with two reviewers, each of whom had more than 10 years’ experience as nephrologists, based on the predictors’ usefulness in clinical practice in maintenance HD patients.

The final selected predictors were as follows: age, vital signs at the time of visit (body temperature, systolic blood pressure, pulse rate, percutaneous oxygen saturation, Glasgow Coma Scale [GCS]), antibiotics use within one week from hospital visit, patient’s complaints (chill, nausea, focal abdominal signs), and laboratory data at hospital visit (white blood cell [WBC] count, platelet count, serum albumin, serum alkaline phosphatase [ALP], C-reactive protein [CRP]).

### Specific Predictors for the HD Population

In addition to the candidate predictors, we identified several further predictors for bacteremia that are specific to the HD population. Variables considered to be related to bacteremia were selected by referencing the existing literature and conducting multivariate regression with clinical expertise. We then selected those variables readily available in a general clinical setting as the specific predictors from among the available variables. Ultimately, non-arteriovenous fistula (non-AVF) use as vascular access [26–29] was identified as a predictor specific to HD patients.

### Statistical Analysis

#### Descriptive statistics

We analyzed the predictors and the outcome as well as other clinical information, including gender, HD vintage, cause of chronic kidney disease (CKD), and pathogens of bacteremia. Continuous and categorical variables are presented as the median (quartile) and number (percentage), respectively.

#### Development of a prediction rule

Among the candidate predictors, the continuous variables were changed to binary variables based on the cut-off value referenced from previous studies. All of the candidate predictors were selected through stepwise backward selection with a p-value<0.05. We then analyzed the cases with complete data available for the selected predictors via this process.

To establish a CPR from the candidate predictors, a regression coefficient-based scoring method was used. First, the ratio based on each β-coefficient relative to a reference that was an intermediate value of the two variables with the smallest β-coefficient was calculated. Then, the ratio was converted to the appropriate integer. The appropriate integer was chosen from integers close to the number ensuring the highest predictive ability for bacteremia. The total score was calculated by summing the scores for each variable.

For further investigation, the modified CPR, developed by adding non-AVF as an HD-specific predictor for bacteremia to the established CPR, was scored to verify the influence of these predictors on the predictive ability for bacteremia. Calibration was performed based on the Hosmer-Lemeshow chi-square statistic [30] and the slope and intercept of the calibration plot [31] for the CPR.

#### Assessment of test performance

To evaluate potential cut-off scores, we computed the sensitivity, specificity, likelihood ratio, positive predictive value, and negative predictive value for the CPR.

#### Validation process

To validate the final model, we used a bootstrapping technique with 200 resamples [32]. The discriminatory ability of the prediction rules was assessed by the area under the receiver operating characteristic curve. All of the statistical analyses were performed using Stata version 13.1 (Stata Corp., College Station, TX, USA).

## Results

### Description of Study Cohort

**Fig 1:**
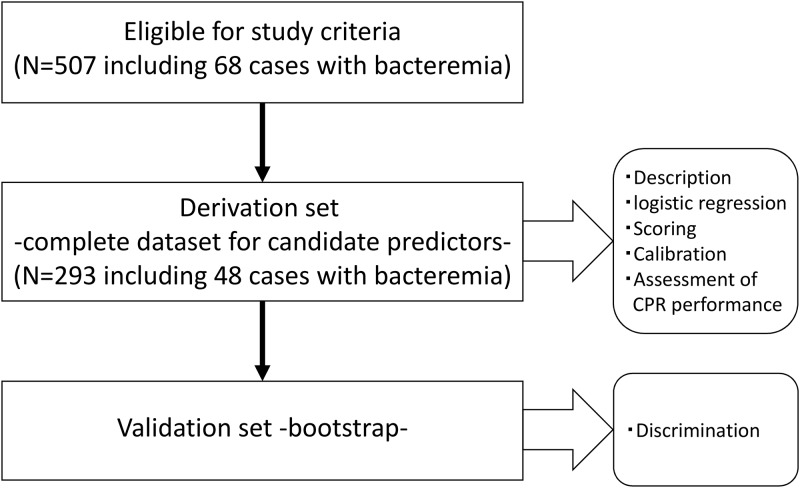
Study flow CPR: clinical prediction rule.

Among 507 participants with 68 cases of bacteremia who met the eligible study criteria, the 293 participants with 48 cases of bacteremia who had a complete dataset for the candidate predictors were analyzed, as shown in Fig 1. Table 1 shows the baseline characteristics of the participants. Of the 293 participants, the median age was 74 years, 66.6% were men, the most frequent cause of chronic kidney disease was diabetic nephropathy (42.0%), the most frequent route of vascular access was arteriovenous fistula (83.6%), the mean HD vintage was 61 months, and 16.4% of patients had taken antibiotics within 1 week prior to hospital visit.

**Table 1:** Baseline characteristics (N = 293).

	Median (quartile or %)		Median (quartile or %)
**Sex**		**Medication**	
Male	195 (66.6)	Steroid use	33 (11.3)
Female	98 (33.5)	Immunosuppressant use	5 (1.7)
**Age (years)**	74 (66, 81)	Antibiotics use within 1 week	48 (16.4)
**Vital signs**		**Symptoms**	
Body temperature (°C)	37.2 (36.6, 38.1)	Chill	13 (4.4)
Systolic blood pressure (mmHg)	136 (114, 160)	Nausea	28 (9.6)
Heart rate (/min)	84 (74, 100)	Focal abdominal sign	29 (9.9)
SpO_2_ (%)	97 (95, 98)	**Causes of CKD**	
(FiO_2_)	0.21 (0.21, 0.21)	Diabetic nephropathy	123 (42.0)
GCS<15	45 (15.4)	Hypertensive nephrosclerosis	61 (20.8)
**HD vintage (months)**	61 (23, 112)	Chronic glomerulonephritis	45 (15.4)
**Vascular access**		Others and unknown	64 (21.8)
AV fistula	245 (83.6)	**Laboratory findings**	
AV graft	28 (9.6)	White blood cell (/μL)	8400 (5900, 11300)
Superficial artery	16 (5.5)	Platelet count (/μL)	14.9 (10.5, 20)
Permanent catheter	4 (1.4)	Albumin (mg/dL)	3.3 (2.9, 3.6)
**History of bacteremia**	31 (10.6)	Alkaline phosphatase (IU/L)	271 (212, 382)
**Comorbidities**		C-reacted protein (mg/dL)	6.1 (1.8, 12.8)
Diabetes mellitus	131 (44.7)	**Bacteremia**	48 (16.3)
Malignancy	33 (11.3)		

SpO_2_: oxyhemoglobin saturation measured by pulse oximetry, FiO_2_: fraction of inspiratory oxygen, GCS: Glasgow Coma Scale, HD: hemodialysis, AV fistula: arteriovenous fistula, CKD: chronic kidney disease

Table 2 shows the pathogens associated with bacteremia in our population. The most frequent pathogen was *Staphylococcus aureus*, accounting for 39.6% (19 cases) of all bacteremia cases. Of these cases, 12 were Methicillin-sensitive *S*. *aureus* (MSSA), while the other 7 were Methicillin-resistant *S*. *aureus* (MRSA). *Klebsiella pnuemoniae* and *Escherichia coli* accounted for 9 and 7 cases, respectively. Among 48 cases with bacteremia, 4 were polymicrobial.

**Table 2:** Pathogens of bacteremia.

Bacteria	N	Bacteria	N
*Staphylococcus aureus*	19	*Ecterococcus faecalis*	1
Methicillin-sensitive *Staphylococcus aureus*	12	*Pseudomonas aerugiosa*	1
Methicillin-resistant *Staphylococcus aureus*	7	*Streptococcus salivarius*	1
*Klebsiella pneumoniae*	9	*Streptococcus pneumoniae*	1
*Escherichia coli*	7	*Streptococcus mutans*	1
Coaglase-negative staphylococcus species	5	*Parabacteroides distasonis*	1
*Clostridium perfringens*	2	*Helicobacter cinaedi*	1
Bacteroides	2	*Anaerobic gram-negative bacilli*	1
*Enterococcus faecium*	2		

### CPR

Stepwise backward selection identified body temperature ≥ 38.3°C, pulse rate ≥ 125/min, CRP ≥ 10 mg/dL, ALP ≥ 360 IU/L, and no antibiotics used within one week before the hospital visit as potential predictors. Table 3 shows the results of regression coefficient-based scoring. Our CPR ultimately included the above 5 variables with 1 point each (total 5 points). The Hosmer-Lemeshow chi-squared statistic for the CPR was 1.99 (p = 0.57), and the calibration slope (intercept) of the CPR was 0.86 (0.01). The sensitivities, specificities, and likelihood ration for possible cut-off scores in prediction rule are shown in Table 4. With a value of 2 set as the cut-off score in the CPR, the sensitivity was 89.6%, the specificity was 51.7%, the positive likelihood ratio was 1.8, the negative likelihood ratio was 0.2, and the percentage of false negatives was 3.8% (5/131).

**Table 3:** Multivariate analysis and scoring.

Selected variables	Β-coefficient	95% CI	p-value	Score
Body temperature ≥ 38.3°C	1.12	0.34, 1.91	<0.01	1
Heart rate ≥ 125 /min	1.12	0.01, 2.22	0.04	1
CRP ≥ 10 mg/dL	1.31	0.60, 2.01	<0.01	1
ALP > 360 IU/L	1.05	0.35, 1.74	<0.01	1
No prior ABx within 1 w	1.3	0.15, 2.45	0.03	1

CRP: C-reactive protein, ALP: alkaline phosphatase, ABx: antibiotics, 95% CI: 95% confidence interval

**Table 4:** Assessment of test performance.

Cutoff	Total	Bacteremia	Sensitivity (95% CI)	Specificity (95% CI)	LR+ (95% CI)	LR- (95% CI)	PPV (95% CI)	NPV (95% CI)
≥1	278	48	100 (92.6, 100)	6.1 (3.5, 9.9)	1.1 (1, 1.1)	0	17.3 (13, 22.2)	100 (78.2, 100)
≥2	162	43	89.6 (77.3, 96.5)	51.4 (45, 57.8)	1.8 (1.6, 2.2)	0.2 (0.1, 0.5)	26.5 (19.9, 34)	96.2 (91.3, 98.7)
≥3	54	22	45.8 (31.4, 60.8)	86.9 (82.1, 90.9)	3.5 (2.3, 5.5)	0.6 (0.5, 0.8)	40.7 (27.6, 55)	89.1 (84.5, 92.8)
≥4	9	5	10.4 (3.5, 22.7)	98.4 (95.9, 99.6)	6.4 (1.8, 22.9)	0.9 (0.8, 1)	55.6 (21.2, 86.3)	84.9 (80.2, 88.8)
≥5	0	0						

LR+: positive likelihood ratio, LR-: negative likelihood ration, PPV: positive predictive value, NPV: negative predictive value, 95% CI: 95% confidence interval

Next, as shown in Table 5, the modified CPR including non-AVF as the HD-specific predictor was scored. The Hosmer-Lemeshow chi-squared statistic for the modified CPR was 2.12 (p = 0.71). The calibration slope and intercept of the CPR were 0.97 and -0.02, respectively.

**Table 5:** Multivariate analysis and scoring for the modified CPR.

	Variables	Β-coefficient	95% CI	p-value	Score
Established CPR + non-AVF	Body temperature ≥ 38.3°C	1.12	0.34, 1.91	<0.01	1
	Heart rate ≥ 125/min	1.12	0.01, 2.22	0.04	1
	CRP ≥ 10 mg/dL	1.31	0.60, 2.01	<0.01	1
	ALP > 360 IU/L	1.05	0.35, 1.74	<0.01	1
	No prior ABx within 1 w	1.3	0.15, 2.45	0.03	1
	Non-AVF	1.3	0.15, 2.45	0.03	1

CPR: clinical prediction rule, CRP: c-reacted protein, ALP: alkaline phosphatase, ABx: antibiotics, 1 w: one week, AVF: arteriovenous fistula

### Internal Validation—CPR

The internal validity of the CPR was verified in the participants extracted via the bootstrap method. The area under the ROC curve (AUC) of the CPR was 0.76 (95% confidence interval [CI]: 0.69, 0.82).

### Internal Validation—Modified CPRs Adding HD-specific Predictors

The internal validity of the modified CPR adding non-AVF was verified in the participants extracted via the bootstrap method. The AUC of the modified CPR was 0.74 (95% CI: 0.67, 0.81).

## Discussion

In this study, we developed a CPR for bacteremia in maintenance HD patients. Although, previous studies have already established CPRs for bacteremia in the general population [11–14], to our knowledge, this is the first prediction rule modified for use in maintenance HD patients. Of the two CPRs, we determined that the CPR not including non-AVF as an HD-specific predictor is the more useful of the pair for clinicians for three reasons. First, the CPR without non-AVF has better diagnostic accuracy with a higher AUC. Second, the study population of this investigation has a much higher proportion of AVF, which is the most common vascular access among Japanese HD patients. As such, a CPR with non-AVF might have low generalizability. Third, a score of ≥2 for the CPR without any HD-specific predictors had high sensitivity and a negative predictive value. We therefore named this CPR the “BAC-HD” (Body temperature ≥38.3°C, Alkaline phosphatase ≥360 IU/L, CRP ≥10 mg/dL, Heart rate ≥125/min, Drug: no prior ABx use within 1 week) score.

A BAC-HD score ≥2 with high sensitivity can be useful for ensuring that bacteremia infections, which are a common and serious issue for HD patients, are not missed. Careful observation can lead to prompt initiation of intravenous antibiotics, which reduces the mortality rate due to infection in dialysis patients.

The BAC-HD score has two major strengths. First, these criteria are considered suitable for use in actual clinical settings, since they include routine clinical data. Specifically, the BAC-HD contains two blood test items—ALP and CRP—that are relatively easy to measure in a Japanese hospital setting; these items provide are nearly as easy to measure as a Complete Blood Count. Furthermore, the present CPR is expected to be used in other facilities besides secondary medical institutions, and the ALP can be measured within 1 h in such facilities. In situations involving HD patients suspected of having bacteremia, although the body temperature, heart rate, history of antibiotic use and CRP are evaluated as a rule in general, the addition of ALP measurement may further support making clinical decisions. Second, the criteria are extremely simple, with only five items, which is few compared with the CPRs for bacteremia in the general population that mostly have more than 10 items. We believe that the simplicity of the BAC-HD score may be attributed to the relatively uniform etiology of bacteremia in HD patients compared with the general population. This simplicity should facilitate the detection of bacteremia in maintenance HD patients and encourage rapid and appropriate decision-making.

In this prediction rule, in addition to the items known to be associated with bacteremia in the general population, we incorporated non-AVF as an HD patient-specific item, which is known to be a strong risk factor for blood stream infections in maintenance HD patients [33] [34, 35]. However, the CPR with non-AVF did not show a superior predictive ability to the original CPR without non-AVF. We attribute this lack of superiority to the markedly high proportion of AVF vascular access in Japan, in contrast to the situation in the United States [36]. Indeed, even in our cohort, the proportion of AVF was 83.6%, indicating little variation, which may have diluted the contribution of non-AVF to bacteremia. Given the recent launch of the “Fistula First” awareness campaign by the National Kidney Foundation to promote the initiation of AVF vascular access in HD induction, we expect AVF access to become mainstream globally. In this context, our prediction rule is considered to be a suitable model for global HD patients in the future.

In the present study, a high ALP level was extracted as an effective predictor. A small observational study further suggested the close relationship between an extremely high ALP level and bacteremia [37]. In that study, the authors speculated that the observed extremely high ALP level was the result of bacteremia-related hepatic dysfunction. ALP is also considered to have an anti-inflammatory effect, given its dephosphorylating and lipopolysaccharide (LPS) detoxifying activity [38–40]. However, LPS is produced by gram-negative bacteria; as such, this etiology for ALP elevation cannot explain the elevation observed in HD patients with bacteremia, as the pathogens in this population are typically *not* gram-negative. Further investigations will therefore be needed to clarify the detailed etiology of ALP elevation in bacteremia among HD patients.

### Limitations

Several limitations to the present study warrant mention. First, given the relatively large size and educational function of the facilities used as the study sites, our rule may not be able to be used at other facilities with different roles in the region. Future studies should conduct external validation of our rule in different settings. Second, because we used a complete dataset for the analysis, subjects with a relatively mild clinical presentation without a detailed history or laboratory test findings were excluded. However, since the measured items in this study were not particularly special and are routinely measured in cases of suspected bacteremia, we believe that such exclusion did not strongly influence the distribution of the severity and comorbidities in the study population. Third, because this was a retrospective cohort study, we cannot deny uncertainty in the data extracted from the medical records. Validation studies should be conducted with a prospective design. Fourth, our sample population was relatively small. However, the required number of outcomes was calculated to be 50 when performing multivariate logistic analyses using five explanatory variables, and we almost met this threshold. Fifth, in the present study, given that we did not evaluate the reasons blood was drawn for culture, we cannot precisely determine the nature of HD patients, particularly feverish patients, who did not undergo blood culture. However, because clinical judgment on this point is often impossible to predict, we believe this lack of consideration actually increases the generalizability, as stated in the preceding paper, which developed a clinical prediction rule for bacteremia in the general population. Sixth, cases with undetectable bacteremia (blood culture-negative) might have existed, which is considered a limitation of blood culture. A new gold standard method for diagnosing bacteremia is needed. Seventh, this CPR is not strictly a predictor because it contains “No prior ABx within 1 week”, which increases the positivity of blood culture. However, we allowed the inclusion of this criterion for the following three reasons: A) If the CPR had been created only for subjects without prior ABx within 1 week, the discrimination ability would have been inferior to that of the BAC-HD score (data not shown). B) Since participants with prior ABx within 1 week were relatively common in the present study (16.4% in this cohort), their exclusion might have reduced the generalizability. C) Given the possibility of bacteremia in participants even with prior ABx, careful observation and treatment likely strongly benefited participants. However, we must recognize the limitations of this score with respect to the low exclusion accuracy in subjects with BAC-HD score <2 who have used ABx in the past week and make any clinical judgments carefully. Finally, in the present study, we were unable to compare the direct discrimination ability between CPRs for the general population and the BAC-HD score because of a data shortage. However, unlike the CPR in the general population, the BAC-HD score does not include items that greatly vary depending on the timing of hemodialysis. Therefore, we believe that the validity of the present CPR for HD patients will likely be higher than CPRs for the general population.

## Conclusion

We developed a simple clinical prediction rule for bacteremia in maintenance HD patients. We expect that using this rule will facilitate the early detection, early treatment, and improvement of prognosis of bacteremia in HD patients.

## Supporting Information

S1 Dataset The anonymous dataset. (XLSX)
