# Correction: Development and Validation of a Clinical Prediction Rule for Bacteremia among Maintenance Hemodialysis Patients in Outpatient Settings

**DOI:** 10.1371/journal.pone.0181800

**Published:** 2017-07-17

**Authors:** Sho Sasaki, Takeshi Hasegawa, Hiroo Kawarazaki, Atsushi Nomura, Daisuke Uchida, Takahiro Imaizumi, Masahide Furusho, Hiroki Nishiwaki, Shingo Fukuma, Yugo Shibagaki, Shunichi Fukuhara, on behalf of Japanese investigatOrs with Innovative Network for Kidney Disease: JOINT-KD

There are errors in the penultimate sentence of the Results section under the subheading “CPR.” The correct sentence is: The Hosmer-Lemeshow chi-squared statistic for the modified CPR was 1.84 (p = 0.39).

There are errors in Table 5. The results of the multivariate analysis and scoring for the modified CPR are incorrect in the β-coefficient, the 95% confidence interval, and the p-value columns. Please see the correct Table 5 and its caption below.

**Table 5:** Multivariate analysis and scoring for the modified CPR.

	Variables	B-coefficient	95% CI	p-value	Score
Established CPR + non-AVF	Body temperature ≥ 38.3°C	1.14	0.34, 1.94	<0.01	1
	Heart rate ≥ 125/min	1.12	0.01, 2.23	0.05	1
	CRP ≥ 10 mg/dL	1.30	0.60, 2.00	<0.01	1
	ALP > 360 IU/L	1.05	0.35, 1.74	<0.01	1
	No prior ABx within 1 w	1.29	0.14, 2.43	0.03	1
	Non-AVF	0.18	-0.74, 1.10	0.70	1

CPR: clinical prediction rule, CRP: c-reacted protein, ALP: alkaline phosphatase, ABx: antibiotics, 1 w: one week, AVF: arteriovenous fistula

## References

[1] Sasaki S, Hasegawa T, Kawarazaki H, Nomura A, Uchida D, Imaizumi T, et al (2017) Development and Validation of a Clinical Prediction Rule for Bacteremia among Maintenance Hemodialysis Patients in Outpatient Settings. PLoS ONE 12(1): e0169975 https://doi.org/10.1371/journal.pone.0169975 28081211 10.1371/journal.pone.0169975 PMC5231279

